# Dynamic Approach to Space and Habitat Use Based on Biased Random Bridges

**DOI:** 10.1371/journal.pone.0014592

**Published:** 2011-01-26

**Authors:** Simon Benhamou

**Affiliations:** CEFE, CNRS, Montpellier, France; University of Utah, United States of America

## Abstract

**Background:**

Although habitat use reflects a dynamic process, most studies assess habitat use statically as if an animal's successively recorded locations reflected a point rather than a movement process. By relying on the activity time between successive locations instead of the local density of individual locations, movement-based methods can substantially improve the biological relevance of utilization distribution (UD) estimates (i.e. the relative frequencies with which an animal uses the various areas of its home range, HR). One such method rests on Brownian bridges (BBs). Its theoretical foundation (purely and constantly diffusive movements) is paradoxically inconsistent with both HR settlement and habitat selection. An alternative involves movement-based kernel density estimation (MKDE) through location interpolation, which may be applied to various movement behaviours but lacks a sound theoretical basis.

**Methodology/Principal Findings:**

I introduce the concept of a biased random (advective-diffusive) bridge (BRB) and show that the MKDE method is a practical means to estimate UDs based on simplified (isotropically diffusive) BRBs. The equation governing BRBs is constrained by the maximum delay between successive relocations warranting constant within-bridge advection (allowed to vary between bridges) but remains otherwise similar to the BB equation. Despite its theoretical inconsistencies, the BB method can therefore be applied to animals that regularly reorientate within their HRs and adapt their movements to the habitats crossed, provided that they were relocated with a high enough frequency.

**Conclusions/Significance:**

Biased random walks can approximate various movement types at short times from a given relocation. Their simplified form constitutes an effective trade-off between too simple, unrealistic movement models, such as Brownian motion, and more sophisticated and realistic ones, such as biased correlated random walks (BCRWs), which are too complex to yield functional bridges. Relying on simplified BRBs proves to be the most reliable and easily usable way to estimate UDs from serially correlated relocations and raw activity information.

## Introduction

**Figure 1:**
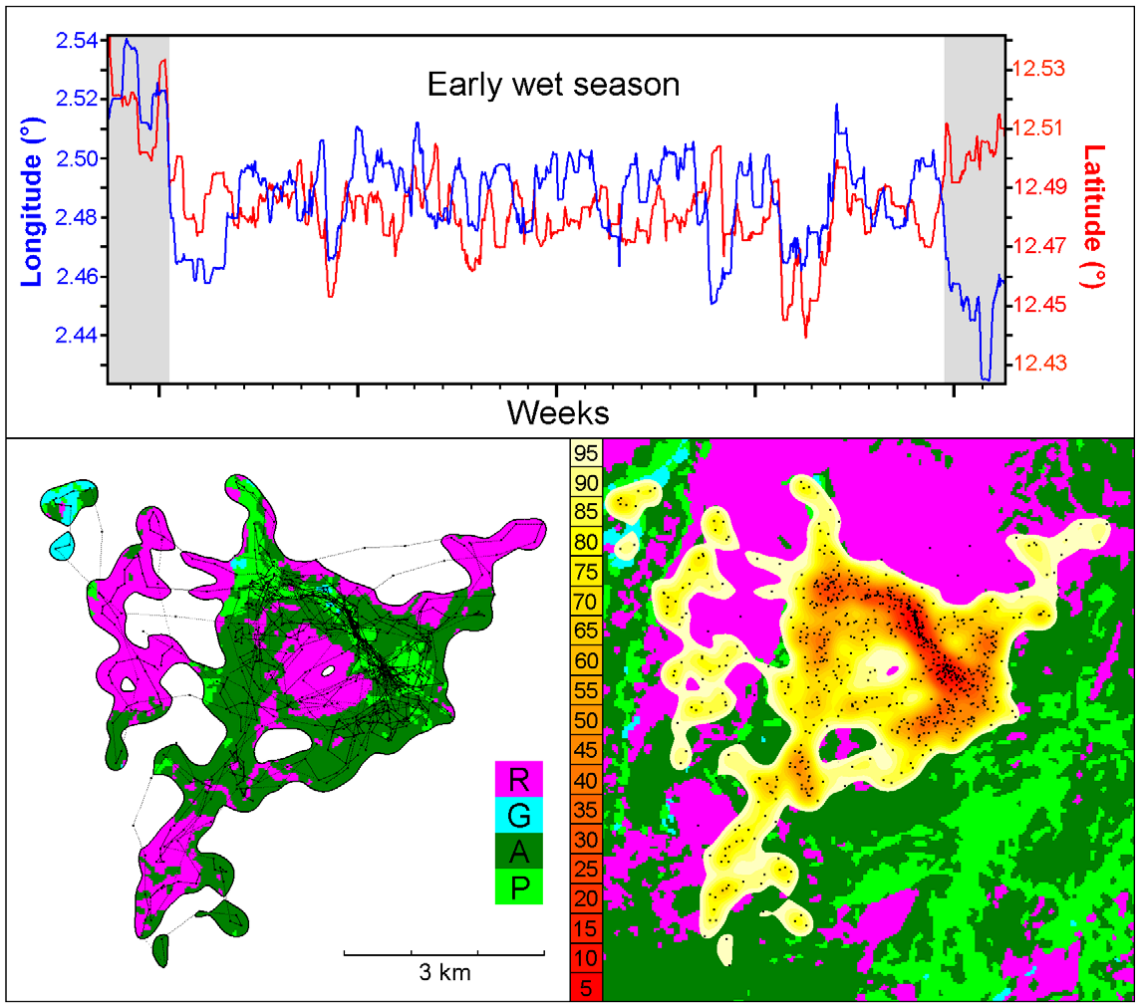
Utilization Distribution (UD) of an African buffalo herd for a 4-week stationary period computed using simplified BRBs (σ*_min_* = 100 m, *D* = 440 m^2^/min and *T_max_*>30 min) through the MKDE method (*h_min_* = σ*_min_* and *h_max_* = (σ^2^ *_min_*+*DT_max_*/2)^0.5^). The top panel shows how the period considered (early wet season, indicated by the white background) was delineated by marked and durable changes in mean or variance of longitude or latitude (computed over a few days in a sliding window). The bottom left panel shows the herd movement (big dots represent GPS relocations recorded at 30-min intervals and tiny dots locations interpolated along track segments at 1-min activity intervals) and the different habitat types available within 95% UD cumulative frequency isopleths (R: Rocky grounds, G: Forest galleries, P: Perennial grasses, A: Annual grasses). The bottom right panel shows the GPS relocations (black dots) and UD cumulative frequencies up to 95% (the colour attributed to a given percentage *p* applies to areas comprised between *p* and *p*–5% isopleths).

Habitat selection is a dynamic process during which a moving animal chooses the habitat patches it visits and the time it spends within each of them. However, even in recent studies involving individuals tracked at a relatively high fix rate using global positioning system (GPS), movement information provided by serial correlation between successive relocations has largely been ignored: habitat use is most often estimated statically as if these relocations were unlinked [1]–[3]. Methods taking relocation history into account are nevertheless emerging [4]. The most direct way to develop a dynamic approach to space and habitat use obviously rests on movement analysis, which highlights how animals can spend more time within preferred habitat patches at each visit [5]–[7] and come back to these patches more frequently [8]. Nevertheless, by integrating the times spent by an animal in the various parts of its home range (HR) over the long term, the utilization distribution (UD) can provide effective complementary information about habitat use [9]. Although they are commonly computed through a static approach involving location-based kernel density estimations (LKDE) [10], [11] over arbitrary periods, UDs can be usefully tackled in a dynamic framework by identifying sub-annual HRs based on stationary phases rather than on proxies of seasonal changes [12] (example in Fig. 1) and by taking advantage of movement and activity information to improve biological relevance of space use estimates. The present paper focuses on this second stage that involves the computation of movement-based rather than location-based UDs.

Contrary to the classical LKDE method, which focuses on the density of presumably unlinked relocations [11], movement-based methods consider the activity times spent between pairs of successive relocations. Two methods of this type have been proposed. The first is based on Brownian bridges (BBs) [13], [14], while the other rests on empirical movement-based kernel density estimation (MKDE) [15]. Paradoxically, in assuming that movements are purely and constantly diffusive (although conditioned by the set of relocations), the BB method denies the existence of HR and habitat preferences, which involve stationary space use patterns [16]–[19] at large scales and changes in diffusion (through changes in speed and sinuosity [20]) and memory-based reorientations [19], [21], [22] at small scales. It might be argued that basic HR behaviour may result from a few rebounds of a diffusive movement at HR boundaries (as for gas molecules in a tank), and habitat preferences may be integrated simply through changes in the diffusion coefficient [23]. However, an advection process is required to allow for more realistic HR behaviour [16]–[19] based on frequent reorientations towards preferred areas, and changes in advection strength can allow for changes in space use intensity much more efficiently than changes in diffusion [21], [24]. On the other hand, the MKDE method is implicitly assumed to be able to deal with any type of movement an animal may perform in its HR, but lacks theoretical foundations. Here I show that using elementary advective-diffusive bridges instead of purely diffusive BBs is the most reliable and easily usable means to outline active UDs from serially correlated relocations and raw activity data. This approach solves theoretical inconsistencies of the BB method and provides sound theoretical foundations for the MKDE method, which turns out to be a useful way to implement such UD computations.

Before entering the core of the subject, let us introduce some basic variables. Consider a GPS tracked animal, whose raw activity (vs. resting) was monitored continuously by a sensor detecting head movements. The *i* ^th^ track segment, linking successive relocations **z** *_i_*_-1_ = (*x_i_*_-1_, *y_i_*_-1_) and **z** *_i_* = (*x_i_*, *y_i_*) is characterised by length *L_i_* = ||**z** *_i_*–**z** *_i_*_-1_||, recording time interval *T_R_*(*i*), proportion of activity time *P_i_* and thereby activity time *T_i_* = *P_i_T_R_*(*i*). Hereafter, the time *t* ∈ [0, *T_i_*] will refer to the activity time elapsed from the previous relocation (resting time is ignored; see rationale in [15]). For simplicity, the track segment rank index *i* will be omitted when unnecessary: any two successive relocations will be referred to as **z**_0_ = (*x*_0_, *y*_0_) and **z** *_T_* = (*x_T_*, *y_T_*).

## Methods

### Biased Random Bridges

Call *f_W_*(**z**, *t* | **z**_0_) the probability density function (PDF) of getting an animal at any location **z** = (*x*, *y*) at a given time *t*>0 knowing that it starts at location **z**_0_ at time *t* = 0 and performs a biased random walk (BRW) with speed *s*. A BRW is a discrete-step walk whose successive movement directions θ are drawn independently from each other in an angular distribution with mean φ, corresponding to the preferred moving direction, and concentration parameter *c* = *E*[cos(θ–φ)] (with *c* = 0, a BRW downgrades to Brownian motion). This advection-diffusion model [20], [24] generates both a drift **v** = [*v* cos(φ), *v* sin(φ)] of the mean location, **μ**_w_(*t*) = **z**_0_+**v** *t*, with speed *v = *||**v**|| = *sc* in direction φ, and an anisotropic diffusion, involving a collinear (*D*_φ_) and an orthogonal (*D*_φ+π/2_) diffusion coefficient. The degree of anisotropy depends on the shape and concentration parameter of the angular distribution and on the movement speed variability [25]. The marginal variance on any arbitrary axis with orientation α, including *X* (α = 0) and *Y* (α = π/2) axes, is 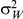
(***t***)_α_ = 2*D*_α_ ***t***, where *D*_α_ = [*D*_φ_+tan^2^(φ–α)*D*_φ+π/2_]/[1+tan^2^(φ–α)] is the diffusion coefficient along this axis. The central limit theorem warrants that f*_W_*(**z**, *t* | **z**_0_) converges (with a speed inversely related to *c*) towards an elliptical bivariate Gaussian PDF, with drift direction φ corresponding to either the major (*D*_φ_>*D*_φ+π/2_) or minor (*D*_φ+π/2_>*D*_φ_) axis:
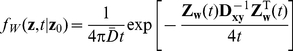
(1)with **Z_w_**(*t*) = **z**–**z**_0_–**v** *t* (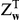 is the transpose of **Z_w_**)

where 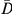 = (*D*_φ_ *D*_φ+π/2_)^0.5^ = [*D*_0_ *D*_π/2_(1–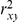)]^0.5^ is the mean (isotropically equivalent) diffusion coefficient and **D_xy_** is the diffusion matrix, based on the diffusion coefficients along the *X* and *Y* axes, *D*_0_ and *D*_π/2_, and correlation *r_xy_* = tan(φ)(*D*_0_ –*D*_π/2_)/[(*D*_0_ *D*_π/2_)^0.5^(1–tan^2^(φ)].

Consider now that the animal is relocated at location **z** *_T_* at time *t* = *T*. Similarly to a BB, a biased random bridge (BRB) is built up by integrating over time (between 0 and *T*) the PDF *f_B_*(**z**, *t* | **z**_0_, **z** *_T_*) of getting the animal at any location **z** at a given time *t*<*T* conditioned by both starting (**z**_0_) and ending (**z** *_T_*) locations: *g_B_*(**z** | **z**_0_, **z** *_T_*) = 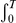 *f_B_*(**z**, *t* | **z**_0_, **z** *_T_*)d*t*/*T*. The movement performed from **z** to **z** *_T_* does not depend on the movement previously performed from **z**_0_ to **z**. The PDF of getting the animal at location **z** *_T_* at time *T*, knowing that location at time *t* is **z**, is therefore equal to *f_W_*(**z** *_T_*, *T*–*t |* **z**). Based on conditional probability formula, it is easy to show that *f_B_*(**z**, *t* | **z**_0_, **z** *_T_*) can be computed as:
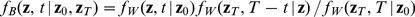
(2)


By combining Eqs (1) and (2) and re-arranging terms, the asymptotic expression of *f_B_*(**z**, *t* | **z**_0_, **z** *_T_*) is shown to correspond to an elliptical bivariate Gaussian PDF, with the same eccentricity and major axis orientation as *f_W_*(**z**, *t* | **z**_0_):
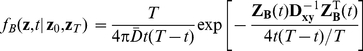
(3)with **Z_B_**(*t*) = **z**–**z**_0_–(**z** *_T_*–**z**_0_)*t*/*T* (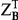 is the transpose of **Z_B_**)

The mean location, **µ_B_**(*t*) = **z**_0_+(**z** *_T_*–**z**_0_)*t*/*T*, slides from **z**_0_ to **z** *_T_* with constant speed *L*/*T.* The marginal variance on any arbitrary axis with orientation α is 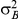
(*t*)_α_ = 2*D*_α_ *t*(1–*t*/*T*). It is therefore null at times *t* = 0 and *t* = *T*, and takes its maximum value, equal to *D*_α_ *T*/2, at time *t* = *T*/2, irrespective of the bridge length *L*. At short times, *f_W_*(**z**, *t* | **z**_0_) has a symmetry axis corresponding to the drift direction but not necessarily a symmetry centre [25]. The additional constraint of being at location **z** *_T_* at time *T* should force *f_B_*(**z**, *t* | **z**_0_, **z** *_T_*) to have a symmetry centre (although not necessarily to be Gaussian), so that its main asymptotic properties should remain (at least approximately) valid at short times.

The advection process affects both the orientation of a BRB, which tends to align in the drift direction, and its shape, as a stronger advection involves a longer and lower bridge on average (the time-integrated PDF *g_B_*(**z** | **z**_0_, **z** *_T_*) is inversely proportional to the bridge length *L*, with *E*(*L*)>*vT*). The mathematical expression of BRBs depends however only marginally (through the matrix diffusion) on the drift characteristics, which do not affect the expected value **µ_B_**(*t*) or the mean diffusion coefficient 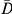. They only affect the degree and orientation of diffusion anisotropy. In theory, these two parameters need to be known to compute BRBs, but they usually cannot be estimated in practice. As they have only a weak influence, diffusion anisotropy can be neglected. For this purpose, BRWs are approximated as Brownian walks (with diffusion coefficient *D*) on which a drift **v** is appended. In this simplified formulation, *f_B_*(**z**, *t* | **z**_0_, **z** *_T_*) converges quickly towards a circular bivariate Gaussian PDF, fully independent of the drift **v** (*D*_α_ = *D* for any axis orientation α, **D_xy_** = *D* **I_2_** where **I_2_** is the 2x2 identity matrix). There is therefore no need to explicitly know the drift characteristics to compute simplified BRBs, which rest on exactly the same PDFs as BBs (**v = **0).

In both standard BRBs (at large times *T*) and simplified BRBs (including BBs), the expected value *E*[δ^2^(*t*)] = 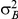
(*t*)_0_+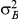
(*t*)_π/2_ of the squared distance δ^2^(*t*) = ||**z** *_t_*–**µ_B_**(*t*)||^2^ between any location **z** *_t_* at time *t* and its expected value **µ_B_**(*t*) is equal to 2*t*(1–*t*/*T*)(*D*_0_+*D*_π/2_). Three properties are noteworthy: (1) *E*[δ^2^(*t*)] takes its maximum value at time *t* = *T*/2; (2) this maximum value is proportional to *T*; (3) the ratio *E*[δ^2^(*t*)]/*E*[δ^2^(*T*/2)] is equal to 4*t*/*T*(1–*t*/*T*) for a given *T* value. Computer simulations showed that, at short times *T*, the first and second properties still hold true for standard BRBs, but the function *E*[δ^2^(*t*)]/*E*[δ^2^(*T*/2)] vs. *t*/*T* tends to be a little bit skewed. This result confirms that, despite differences in diffusion anisotropy and short-time behaviour, simplified BRBs act as valid proxies for standard BRBs.

### Space use estimates based on simplified Biased Random Bridges

It was cleverly proposed [13], [14] that local space use density could be computed from bridges linking every couple of successive relocations as the time-weighted average of their respective time-integrated PDFs: *u*(**z**) = Σ*_i_T_i_ g_B_*(**z** | **z** *_i_*_-1_, **z** *_i_*)/Σ*_i_ T_i_*. The local contribution of each bridge to the UD is thus nicely assumed to be proportional to the time spent by unit length *T_i_*/*L_i_*. However, these pioneering studies considered only bridges that are Brownian and characterized by the same diffusion coefficient, and thereby should be relevant only for animals wandering (no HR) in a uniform environment (purely random resource distribution). The use of simplified BRBs in place of BBs, involving an advective-diffusive instead of a purely diffusive movement process, should provide a sound theoretical basis to compute HR space use estimates. Indeed, not only do changes in advection direction φ allow for critical reorientations towards preferred HR areas but changes in advection strength can further allow for changes in space use intensity more efficiently than changes in diffusion because the bridge shape is more sensitive to the drift speed *v* than to the diffusion coefficient *D*. As BBs and simplified BRBs rest on the same PDF, methods formerly developed to compute BB-based UDs can nevertheless provide valid estimates when some conditions are fulfilled (see below).

A key point in BRBs is that the advection component (and possibly the diffusion coefficient) is allowed to change freely (both in terms of direction and strength) between bridges, but should nevertheless remain constant during each of them (Eqs 1–3 are not valid otherwise). It is therefore necessary to set an upper time threshold *T_max_* to warrant homogeneous movements (i.e. no marked drift or diffusion changes) between successive relocations: track segments that are longer in time are dismissed from UD computation. The introduction of this time threshold results in an overall upper limit for the BRB movement variance 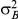
(*t*), obtained at time *t* = *T*/2 for *T* = *T_max_*: 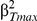 = *DT_max_*/2. In contrast, there is no theoretical reason to include this time constraint in the BB method, as it *a priori* assumes purely diffusive movements characterised by a diffusion coefficient that remains constant for the whole tracking period.

A last question concerns the space use intensity to attribute to recorded locations. To circumvent the infinite values taken by *f_B_*(**z**, *t* | **z** *_i_*_-1_, **z** *_i_*) at times *t* = 0 and *t* = *T_i_* and to take the recording noise into account, it was proposed [13], [14] to give BBs a total variance, 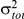
(*t*)*_i_*, written as the sum of the time-weighted recording noise variance ε^2^ and movement variance 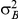
(*t*)*_i_*, 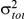 (*t*)*_i_* = ε^2^[*t* ^2^+(*T_i_*–*t*)^2^]/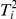 +2*Dt*(1–*t*/*T_i_*). If this solution was applied to BRBs, the total variance would paradoxically take its lowest rather than highest value at mid-distance (*t* = *T_i_*/2) when the recording noise is large (ε^2^>*DT_i_*) and the animal moves quite straight (involving a strong drift and low diffusion). Furthermore, when the recording noise is practically negligible with respect to patch sizes, as occurs with current GPS tracking, it seems preferable to incorporate a relocation variance that is associated to animal behaviour instead of recording device noise [15]. Indeed, a GPS fix should be considered a punctual sample of the possible locations at which the animal may be observed at that time, given its current motivational state and history. Even if the recording noise is low, the relocation variance should therefore be large enough to encompass potential locations occurring in the same habitat patch as the recorded location. This relocation variance should represent the minimum value of the total variance even for an animal moving straight for a while and will therefore be referred to as 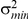. It may be assumed either to have a constant weight in the total variance or to progressively merge with the movement component 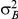
(*t*)*_i_*. This leads to the respective expressions of the total variance:
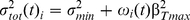
(4a)

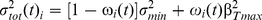
(4b)with ω*_i_*(*t*) = 4*t*(1 –*t*/*T_i_*)/*T_max_*. Although Eq. (4b) is more intuitively appealing, it can be applied only for *D*>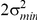/*T_max_*, and the result it provides marginally depends on the chosen value of *T_max_*, contrary to Eq. (4a), which warrants full independence. Space use density at any location **z**, *u*(**z**), can then be computed from the time-weighted contributions of the simplified BRBs associated to the *N_S_* track segments of the path: 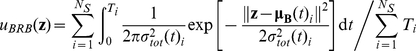
(5)with **µ_B_**(*t*)*_i_* = **z** *_i_*_-1_+(**z** *_i_*–**z** *_i_*_-1_)*t*/*T_i_* and *T_i_* reset to 0 for any track segment lasting more than *T_max_*.

### Computing simplified BRB-based UDs through location interpolation

In the MKDE method [15], movement information provided by serially correlated locations is incorporated through location interpolation in the otherwise classical LKDE framework [10], [11]. Although it was developed on empirical grounds, I show here that the MKDE method turns out to be a useful way to compute simplified BRB-based UDs.

The method consists in dividing the *i* ^th^ track segment in *n_i_ = * round(*T_i_*/τ) intervals, where τ is a time constant, by interpolating *n_i_*–1 equidistant locations along it. In this way, contributions to the UD of the successive relocations distribute preferentially in the local movement direction rather than uniformly in any direction (as occurs in the LKDE method), and the local density of interpolated locations warrants that these contributions are proportional to the times spent per unit length *T_i_*/*L_i_*. The *m* ^th^ location of the *i* ^th^ segment, ζ*_i_*(*m*) = **z** *_i_*_-1_+(**z** *_i_*–**z** *_i_*_-1_)*m*/*n_i_* (with *m* = 0, 1, …, *n_i_*), lies at distance *mL_i_/n_i_* from the previous relocation **z** *_i_*_-1_. It therefore represents the expected animal's location at time *t* = *mT_i_*/*n_i_* in BB and BRB theory. As too large delays in relocation may result in dubious movement information due to a too weak serial correlation, an upper recording time limit, *T_max_*, has been introduced to filter out couples of successive relocations that are not sufficiently serially correlated to warrant that the animal was more likely to be in between them than anywhere in its HR at intermediate times. No locations are therefore interpolated along track segments lasting more than *T_max_*. These segments are also ignored in the BRB method for similar reasons, although expressed in a slightly different way.

A variable smoothing parameter (which acts as a standard deviation in kernel functions) is then attributed to any (interpolated or recorded) location:
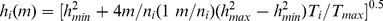
(6)


Its lower limit, *h_min_* (>0.5τ*L_i_*/*T_i_* for all *i* thanks to a small enough τ value) applies to any recorded location (*m* = 0 or *m* = *n_i_*). Its upper limit, *h_max_*, applies to the centre of any track segment lasting *T_max_* for which the animal was always active (*T_i_* = *T_max_*). Thus, *h_i_*(*m*) takes its minimum value at times *t* = 0 and *t* = *T_i_*, and reaches a local maximum (which increases with *T_i_*) at time *t* = *T_i_*/2. Although various equations can give rise to such properties, the particular form of Eq. (6) was designed to be consistent with BB theory [15]. In incorporating an upper time threshold *T_max_*, Eq. (6) turns out to be the discrete time expression of the total standard deviation of simplified BRBs: it can be derived from Eq. (4) by setting σ*_tot_*(*t*)*_i_* = *h_i_*(*m*), *t*/*T_i_* = *m*/*n_i_*, σ*_min_* = *h_min_* and either β*_Tmax_* = [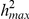
–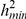]^0.5^ (Eq. 4a) or β*_Tmax_* = *h_max_* (Eq. 4b).

Space use density at any location **z**, *u*(**z**), is then estimated using circular bivariate Gaussian kernel functions centred on locations ζ*_i_*(*m*) with standard deviation *h_i_*(*m*). As the track segments considered (*T_R_*(*i*)≤*T_max_*) are not necessarily contiguous, it is preferable to re-index the whole set of *N_L_* recorded and interpolated locations as ζ*_k_* and associated smoothing parameters as *h_k_* (with *k* = 1, 2, …, *N_L_*) independently of the track segment to which they belong, before computing *u*(**z**) as:
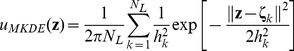
(7)


It eventually turns out that Eq. (7) corresponds to an easy and effective way to work out Eq. (5), which has no analytical solution.

### Diffusion coefficient and drift speed estimations

Consider a triplet of successive relocations **z** *_i_*_-1_, **z** *_i_* and **z** *_i_*_+1_. If **z** *_i_* were missing but the drift **v** remained approximately constant between the previous and next recording times (i.e. *T_R_*(*i*)+*T_R_*(*i*+1)≤*T_max_*), the expected animal location at time *t* = *T_i_* would be **µ_B_**(**z** *_i_*) = **z** *_i_*_-1_+(**z** *_i_*_+1_–**z** *_i_*_-1_)*T_i_*/(*T_i_*+*T_i_*_+1_), with associated movement variance 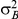
(*T_i_*) = 2*DT_i_T_i_*_+1_/(*T_i_*+*T_i_*_+1_). The expected value of the squared distance 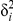 = ||**z** *_i_*–**µ_B_**(**z** *_i_*)||^2^ between actual relocation **z** *_i_* and its expected value **µ_B_**(**z** *_i_*) is therefore *E*(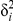) = 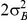
(*T_i_*) = 4*DT_i_T_i_*_+1_/(*T_i_*+*T_i_*_+1_). The diffusion coefficient *D* can then be estimated from the *N_C_* couples of consecutive track segments involved as:
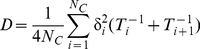
(8)


For safety, this computation should be restricted to movements liable to be globally homogeneous between **z** *_i_*_-1_ and **z** *_i_*_+1_ by dismissing couples with *T_i_*>2*T_i_*_+1_ or *T_i_*<*T_i_*_+1_/2 or *L_i_*>2*L_i_*_+1_ or *L_i_*<*L_i_*_+1_/2. Alternatively, *D* may be estimated using maximum likelihood [14]. There are also two noteworthy cases where *D* should be set to 0: when the animal is immobile (although active) or when it moves straight between relocations (purely advective movements). Both cases may occur simultaneously with browsers eating on shrubs scattered in semi-desertic habitats. The maximum movement variance, 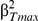 = *DT_max_*/2, can then be used in Eq. (4a) or (4b) to estimate the time-specific PDF *f_B_*(**z**, *t_i_* | **z** *_i_*_-1_, **z** *_i_*) of simplified BRBs associated to track segments lasting less than *T_max_*, and in Eq. (6) to set the maximum smoothing parameter value of the MKDE method: *h_max_* = (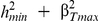)^0.5^ or *h_max_* = β*_Tmax_*, depending on whether the relocation variance is assumed to be constant or to merge progressively within the movement variance.

Habitat-specific diffusion coefficients *D_H_*, estimated by applying Eq. (8) to the couples of consecutive track segments that are fully included in the same habitat type *H*, may be easily incorporated in the MKDE method by attributing habitat-specific *h_max_* values to the various recorded and interpolated locations. Very high fix rates are however required in practice to get sufficiently large samples of couples of consecutive track segments occurring in the same habitat type to reliably estimate *D_H_* coefficients for the habitat types that are scarce or fragmented. Squared distances 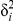 are asymptotically distributed according to a rescaled 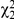 law [20], involving large random errors (coefficient of variation equal to 1). This can lead to noticeable over or underestimations of *D_H_* values when sample sizes are low.

The location **z** *_i_* reached at time *t* = *T_i_* by a simplified BRW obeys a circular bivariate PDF *f_W_*(**z** *_i_*, *T_i_* | **z** *_i_*_-1_) with mean **µ_W_**(*T_i_*) = **z** *_i_*_-1_+**v** *T_i_* and variance 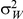
(*T_i_*) = 2*DT_i_*. The expected squared distance (track segment length) is therefore *E*(*L_i_* ^2^) = *v* ^2^ *T_i_* ^2^+4*DT_i_*. Even if the diffusion coefficient is set to a single global value *D*, the drift speed *v* is ever allowed to depend on the habitat type traversed by varying between track segments. The squared drift speed for each habitat type *H* can be estimated from the *N_H_* track segments that are fully encompassed in the habitat type considered (it is obviously much easier to find single track segments than couples of consecutive track segments that are fully encompassed in a given habitat type) as:
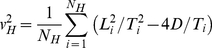
(9)


Less preferred habitat types should be characterised by larger values reflecting fast and oriented transit movements whereas lower values should indicate mainly diffusive movements within patches of highly preferred habitat types. Negative values may even occur because the latter movements are likely to involve habitat-specific diffusion coefficients *D_H_* lower than the global *D* value that has been estimated for the whole environment. More reliable drift speed values should therefore be obtained when reliable habitat-specific coefficients *D_H_* can be used in Eq. 9 instead of a global *D* value.

## Results

Here I illustrate the relevance of the BRB method to estimate habitat use by an African buffalo (*Syncerus caffer*) herd in the early wet season of 2008. This herd inhabited areas close to the south-western bank of the Niger River in W park (see [26] for details). One female was equipped with a GPS collar programmed to acquire fixes at *T_R_* = 30 min intervals. Raw activity (resting vs. non-resting) was continuously monitored over 5-min intervals using a head-movement sensor included in the GPS collar. As ruminant grazers, buffalos move when they eat. Track segments shorter than 50 m were therefore filtered out even when associated to a high proportion of activity time (possibly due to disturbance by flies). Note that, for species that can intensively feed while remaining almost immobile like browsers, such short and active bouts should be kept but given a null diffusion coefficient.

Most buffalo herds in [26] were tracked at 180 min intervals, involving median distances between successive relocations about 10 times smaller than HR diameters. Larger delays resulted in loose serial correlations. Hence, it appears unreasonable to set *T_max_* to a value larger than 180 min. In the present example, 99.7% of the recording intervals lasted 30 min and 0.3% 60 min due to a few missing fixes. Hence, UD estimates (Fig. 1) obtained by setting *T_max_* to any value larger than 30 min will be almost (*T_max_*<60 min) or strictly (*T_max_*≥60 min) identical. Keep in mind however that, to estimate the diffusion coefficient *D*, the drift has to be assumed to be constant during at least two consecutive track segments (i.e. *T_max_*≥60 min).

Given the fragmented structure of the habitat, a reasonable choice for σ*_min_* would have been to set its value to about 50 m for a solitary individual. As the centre of gravity of a herd does not necessarily coincide with the location of the tracked individual, it seems preferable to set σ*_min_* to a larger value, e.g. 100 m. Application of Eq. (8) revealed that the global diffusion coefficient *D* was 440 m^2^/min (β_180_ = 200 m). Habitat-specific *D_H_* values ranged between 220 m^2^/min (β_180_ = 140 m) and 350 m^2^/min (β_180_ = 180 m). Most of these *D_H_* values must be considered with caution, however, because of the small sample sizes (Table 1).

**Table 1:** Habitat-specific diffusion coefficients, preferences and drift speeds.

	Diffusion coefficient	% Habitat availability	% Habitat use	Normalised preference	Drift speed
Rocky grounds	350 (20)	40	18	0.096	16 (75)
Galleries	–– (0)	2	1	0.149	13 (2)
Annuals	320 (61)	47	53	0.237	9 (204)
Perennials	220 (21)	11	28	0.518	6 (86)

Most habitat-specific diffusion coefficients (expressed in m^2^/min) were computed from too few couples of consecutive track segments (sample sizes between parentheses) to be reliable. Both habitat availability (non-weighted proportion of each habitat type) and use (UD-weighted proportion of each habitat type) were computed on the areas encompassed within 95% isopleths (see Fig. 1). Normalised preferences correspond to habitat use/availability ratios subject to unit-sum constraint. Drift speeds values (expressed in m/min) should be considered with extreme caution when computed from only a few track segments.

The results obtained in terms of normalised (between 0 and 1) habitat preferences are quite robust (changes ≤0.01) to the choice of σ*_min_* (50 vs. 100 m) or the way σ*_min_* and β*_Tmax_* are combined (Eq. 4a vs. 4b). Unsurprisingly, the squared drift speed (estimated using Eq. 9) took its highest value in areas belonging to the least preferred habitat type and its lowest value in areas belonging to the most preferred habitat type (Table 1).

## Discussion

### Biological relevance of simplified Biased Random Bridges

Animal movements are best modelled as biased correlated random walks (BCRWs) with adjustable levels of directional bias and directional correlation [20], [24]. With a constant bias direction, BCRWs, as BRWs, are ballistic at long time periods (the net straight line displacement tends to be proportional to the travel length) whereas, with a bias directed toward a central place, they lead to stationary space use patterns [16], [21]. Without any bias, BCRWs reduce to CRWs, which are diffusive walks at long time periods but seem to be ballistic at short ones [20], [24]: their tendency to hold an initial movement direction for a while results in a short term pseudo-drift that is inversely related to their sinuosity. Contrary to Brownian motion, simplified BRWs can therefore represent various types of animal movements at short time periods quite realistically. Using computer simulations, it can be shown however that the expected value of the squared distance δ^2^(*t*) = ||**z**_0_+(**z** *_T_*–**z**_0_)*t*/*T*–**z** *_t_*||^2^ does not present exactly the same properties in CRBs and BRBs: *E*[δ^2^(*t*)] takes its maximum value at time *t* = *T*/2 in both BRBs and CRBs, but this maximum value tends to increase faster than *T* in CRBs, and the function *E*[δ^2^(*t*)]/*E*[δ^2^(*T*/2)] vs. *t*/*T*, which is parabolic in simplified BRBs, tends to be more bell-shaped in CRBs. These differences are sufficiently weak to imply that simplified BRBs constitute a valid approximation of CRBs or BCRBs when relocations are acquired frequently on a regular time basis (so as to minimize inter-bridge variations in time *T*). Simplified BRBs are as simple as BBs to apply, as they can be computed without any knowledge about the direction and strength of the advection. They are thus the only advective-diffusive bridges that are sufficiently simple to be usable in practice, while they remain sufficiently sophisticated to suitably represent various types of movements between known relocations acquired within a relatively high rate.

The introduction of an advection process whose characteristics can change from one bridge to the next allows for the actual abilities of a resident animal to frequently reorientate towards the more attractive areas of its HR (through changes in advection direction) and to locally adapt its space use intensity to the type of habitat it crosses (through changes in advection strength). Simplified BRBs thus provide a functional way to obtain reliable HR space use estimates from GPS fixes that have been obtained at a high rate and raw activity information. Simplified BRB-based UDs can easily be computed using the MKDE method, which can further cope with the presence of obvious boundaries to avoid attributing non-null space use values to inaccessible areas and underestimated values to accessible adjacent areas [15]. Finally, drift speed estimates make it possible to quantify the extent to which higher cumulative space use frequencies in some areas were due to higher habitat-dependent instantaneous space use intensity. Complementary path recursion analyses [8] are required to determine the extent to which preference for these areas was also due to more frequent returns to them. A user-friendly program for computing BRB/MKDE-based UDs (including boundary management and, if a habitat map is available, habitat preferences and drift speed estimations) in a form allowing effortless integration in ArcGIS and R/adehabitat environments [27] is available upon request.

Given the crucial function of advection, the validity of HR space use estimates obtained with the BB method is questionable. As BBs and simplified BRBs involve identical PDFs, the computational procedure on which the BB method rests should be globally valid when animals performs advective-diffusive movements with a locally (within bridge) constant drift instead of purely diffusive ones. In practice, the BB and BRB methods differ primarily by the specification of an upper recording time threshold *T_max_* in the latter, and secondarily by different expressions of the relocation variance. Thus, for an animal tracked with a high fix rate with respect to its usual movement speed and HR size, as in the buffalo example, the BB and BRB methods will provide similar (identical if the same relocation variance was used) UD estimates because all recording time intervals *T_R_* were shorter than any reasonable *T_max_* value. In such cases, the BRB method will not provide better results than the BB method would do, although confidence in their validity can be higher.

### Key role of the upper recording time threshold

In both BB and BRB methods, the expected animal location at intermediate times is assumed to slide along a straight line from one relocation to the next with a constant local speed, involving a homogenous movement process. The key role of the upper recording time threshold *T_max_* is precisely to filter out track segments that are likely to involve marked changes in the preferred moving direction and/or other movement characteristics. There is obviously no reason to introduce such a time threshold when movements are *a priori* assumed to be purely and constantly diffusive. It has been acknowledged, however, that too long delays between successive relocations may be problematic for the BB method because they may involve some orientation process (i.e. advection) towards the HR centre [14]. The identity of BB and simplified BRB equations shows that the existence of an orientation process towards any goal (not limited to the HR centre) does not matter by itself, provided that recording time intervals *T_R_* are small enough to warrant that its characteristics remain approximately constant between successive relocations.

The time threshold *T_max_* prevents the use of the BRB method when animals were tracked with a too low fix rate. If numerous rack segments are longer in time than *T_max_*, the fix data set is then better considered as the output of a point rather than a movement process, and UDs are better estimated using the classical LKDE method. The BB method might still be used in this case, as it incorporates no time threshold, but will provide dubious results. The BRB method is quite flexible when implemented via MKDE as it mechanically generates LKDE-based UD estimates with a constant smoothing parameter equal to *h_min_* (which must be set to an appropriate value for LKDE) whenever all track segments are longer in time than *T_max_*. With animals tracked with a relatively high fix rate, too long track segments may nevertheless still occur because of missing fixes (GPS failures). The BB and BRB methods will provide similar UD estimates when there are no missing fixes, but will increasingly diverge as the fraction of segments longer than *T_max_* increases. The BB method will tend to provide increasingly dubious results because it will estimate erroneous space use frequencies attached to an increasing number of too long segments, whereas the BRB method, which filters them out, will tend to provide increasingly biased results in terms of habitat preferences because GPS failures are often habitat-dependent [28], [29]. However, this bias may be corrected later by re-attributing the activity times initially removed from UD computation to the habitats in which the animal was likely to be. Finally, if the programmed recording time intervals were lower than *T_max_*/2, occasional missing fixes will be replaced by bridge probability estimations with a cost limited to a marginal loss of accuracy.

A general guideline to determine a suitable *T_max_* value consists in estimating the serial correlation between subsampled relocations. Determining whether the correlation level is low enough to consider relocations as being statistically independent is relatively easy [30]. In contrast, it is harder to determine whether the serial correlation between relocation is high enough to provide suitable movement information. In the absence of standardized procedure, one may consider that the serial correlation is sufficiently high when the median distance between successive relocations is much (e.g. 10 times) smaller than the HR diameter. If it is necessary to keep one relocation every *n* to obtain a low serial correlation, the *T_max_* value can be set to *n*–1 times the recording time interval (assuming that data were acquired with a constant recording time interval). Setting *T_max_* to any larger value will lead to identical results if the GPS failure rate is negligible. When an animal cannot be tracked with a high fix rate for extended periods because of electric power constraints, the recording time interval could be set to a reasonable *T_max_* value (e.g., based on a pilot study) if the failure rate is negligible, but a doubled rate will have to be used occasionally to estimate the diffusion coefficient *D*. If GPS failures occur more often, this doubled rate will have to be used routinely.

### Towards a dynamic approach to space and habitat use

Before the GPS era, getting wild animal locations in the field often required manual triangulation, a highly time-demanding task. Wildlife researchers then usually looked at acquiring only the minimum relocation number necessary to obtain reliable HR size estimates. It was then important to check that relocations were statistically independent [30], [31] because a serial correlation indirectly meant that the whole tracking period was too short to let animals move around in their whole HRs. However, serial correlation does not matter by itself in HR estimation [32]–[35], and getting samples of serially correlated relocations that are large enough to be representative of the whole HR use is currently no problem with GPS tracked animals. In this new context, needs are reversed: it has become important to record highly serially correlated relocations so as to obtain effective movement information. Recording raw activity concurrently is also of major importance to distinguish resting and intensive space use [15]. In principle, only activity times matter in bridge computations (resting habitat preferences can be estimated separately from resting locations). If of some interest, however, global rather than active UDs can be computed with a modified version of the BRB method in which resting relocations are kept and given a null diffusion coefficient.

Although it has been applied with apparent success to markedly serially correlated relocations, the LKDE method was initially designed to deal with independent locations. It involves a smoothing parameter which can be fixed or variable, and depends on the global (fixed) or local (variable) location density [10], [11]. The best way to estimate this key parameter in HR studies is still open to discussion [36]. A poor choice is likely to result in unreliable UD estimates [3], [15], [34]. In fact, with serially correlated locations reflecting a movement rather than an independent point process, probability density estimation is not just a matter of location density. Through its MKDE form, the BRB method involves a variable smoothing parameter whose minimum value depends on the habitat grain and maximum value depends on (possibly habitat-specific) movement diffusion coefficient(s). It should therefore provide more biologically relevant UD estimates. By relying on very general movement rules (advection-diffusion), this promising approach stays at the interface between general statistical approaches, which can coarsely describe any location pattern and relate it to habitat covariates [9] but fully ignore the underlying movement processes, and mechanistic approaches [16], [37], which rely explicitly on hypothetical movement processes that may be too specific to apply to a large range of situations. The dynamic approach to space and habitat use based on BRBs should therefore contribute to a renewed foraging theory [38] by bridging the gap between habitat selection and movement ecology studies.
